# Echocardiographic assessment of asymptomatic US Air Force members with early HIV infection

**DOI:** 10.1186/s13104-019-4822-y

**Published:** 2019-11-29

**Authors:** Gadiel R. Alvarado, Courtney R. Usry, Rosco S. Gore, James A. Watts, Jason F. Okulicz

**Affiliations:** 1 Infectious Disease Department, San Antonio Military Medical Center, San Antonio, TX 78234 USA; 2 Cardiology Department, San Antonio Military Medical Center, San Antonio, TX USA

**Keywords:** HIV, People living with HIV, Cardiovascular disease, Echocardiography

## Abstract

**Objective:**

People living with HIV (PLHIV) are at increased risk for cardiovascular disease (CVD) and development of subclinical echocardiographic abnormalities. However, there is scant evidence of the echocardiographic changes that occur shortly after seroconversion. In this study we describe the echocardiographic evaluations of asymptomatic US Air Force members who were diagnosed with HIV infection and evaluated at the San Antonio Military Medical Center between September 1, 2015 and September 30, 2016.

**Results:**

Patients (n = 50) were predominantly male (96%), mostly African American (60%), with a mean age of 28 years. At HIV diagnosis, the mean viral load was 112,585 copies/mL and CD4 count was 551 cells/μL. All were found to have normal left ventricular systolic ejection fraction (EF) and global longitudinal strain (GLS) however evidence of right ventricular dilatation and left ventricular remodeling was observed in 7 (14%) and 13 (26%) patients, respectively. Subgroup analyses showed no significant differences in echocardiographic findings by HIV disease severity or CVD risk factors (p > 0.05 for all).This study suggests that untreated HIV may have a low impact on the development of echocardiographic abnormalities shortly after seroconversion. Longitudinal studies are warranted to determine the optimal CVD risk assessment strategies for PLHIV.

## Introduction

Over the past 2 decades, advances in HIV care has improved the life expectancy of people living with HIV (PLHIV). And as expected cardiovascular disease (CVD) has become the main cause of morbidity and mortality among the aging HIV-infected population [1]. Current CVD prevention strategies for PLHIV mainly consist of minimizing traditional risk factors. However increasing evidence suggests HIV infection alone leads to more accelerated development of CVD despite traditional risk factor modifications [2]. For this reason, many clinicians have identified a need for research in early detection, prevention and risk reduction of CVD among PLHIV [3].

Despite recognition of HIV infection as a risk factor for development of CVD, the pathophysiology of HIV-induced CVD has not been fully elucidated [4]. This is particularly true for the early infection period, as most of the data currently available is from chronically infected populations or in those with unknown duration of HIV infection [5, 6]. We describe the findings of non-invasive cardiovascular health evaluations performed during the initial medical assessment of asymptomatic US Air Force (USAF) members with early HIV infection prior to the initiation of ART.

## Main text

### Methods

#### Patients

The SAMMC Institutional Review Board approved a retrospective analysis of all ART-naïve USAF members with newly diagnosed HIV evaluated between September 1, 2015 and September 30, 2016. All active duty USAF members are required to have annual HIV screening and those diagnosed with HIV infection have an initial medical evaluation at the San Antonio Military Medical Center (SAMMC). The initial visit consists of a multidisciplinary evaluation to determine fitness for military duty by an infectious disease physician which includes a comprehensive cardiovascular risk assessment and structural evaluation by transthoracic echocardiogram (TTE).

All the patients evaluated during the period met inclusion criteria for the study.

#### HIV disease characteristic evaluation

Electronic medical record data were used to classify patients by HIV disease severity and estimated duration of HIV infection. The estimated date of seroconversion was defined as the midpoint between the last negative HIV test and the first positive HIV test. The estimated duration of HIV infection at initial evaluation was defined as the time from the estimated date of seroconversion to the initial evaluation. In addition to HIV-related data, records were also examined for family history, active or prior tobacco use, obesity, hypertension, diabetes, dyslipidemia, and other CVD risk factors.

#### Echocardiographic evaluation

Echocardiographic measurements of left ventricular systolic ejection fraction (EF) and global longitudinal strain (GLS) were used to determine normal cardiovascular function defined as > 51% and − 15.9% to − 22.1%, respectively using the Phillips iE33 ultrasound device using biplane method of disc as well as onboard strain and 3D analysis software. Diastolic function was assessed by measurement of medial and lateral mitral annular motion, mitral valve and pulmonary venous pulse wave Doppler in accordance with current American Society of Echocardiography guidelines [7]. Left ventricular geometry was determined from 2D-guided linear measurement of the parasternal long axis view. The interventricular septum, left ventricular and posterior wall diameters were measured at end diastole. Left ventricular remodeling was defined as relative wall thickness of > 0.42 and hypertrophy was defined as an indexed mass of > 95 g/m^2^ for females and > 115 g/m^2^ for males [8]. Right ventricular size and function was obtained by measuring end-diastolic and end-systolic areas from a right ventricular focus apical view with [8]. A right ventricular fractional area change of < 35% was considered systolic dysfunction and an indexed diastolic area of > 12.6 cm^2^/m^2^ for male and > 11.5 cm^2^/m^2^ for females was considered dilated. Other incidental TTE abnormalities including right ventricular (RV) dilatation, left ventricular remodeling and diastolic dysfunction were also described.

#### Statistical analysis

Variables including CD4 cell count, serum HIV viral load (VL), high-sensitivity C-reactive protein (hs-CRP) and, N-terminal pro-brain natriuretic peptide (NT-proBNP) levels, estimated duration of HIV infection and traditional cardiovascular disease risk factors were analyzed for potential association with TTE abnormalities using Chi squared and Fisher’s exact tests.

### Results

A total of 50 patients were evaluated during the study period. Patients were predominantly male (96%) with a mean age of 28 years ± 7.14 at HIV diagnosis. The mean CD4 count at HIV diagnosis was 551 cells/μL ± 225.1; 20 (40%) patients had a CD4 cell count < 500 cells/μL and 2 (4%) patients had a diagnosis of AIDS by CD4 criteria (< 200 cells/μL and/or CD4% < 14%). A total of 15 (30%) patients had a high VL at HIV diagnosis defined as > 100,000 copies/mL. The mean time from last HIV negative test to first positive HIV test was 530 days ± 265.2 and the mean duration of HIV infection prior to initial evaluation was 304 days ± 129.4. None of the patients had evidence of opportunistic infections or coinfection with hepatitis B or C virus. During the initial cardiovascular assessment, 10 (20%) individuals were active smokers, 4 (8%) had undiagnosed hypertension, and 5 (10%) had dyslipidemia. The mean hs-CRP and NTpro-BNP levels were 1.6 mg/dL ± 2 (low risk < 2 mg/dL) and 19.6 pg/mL ± 39.7 (normal range 0–51 pg/mL), respectively. All patients were found to have normal systolic ejection fraction (63.4% ± 6) and normal GLS (− 20.3% ± 2.7). The  average inter-ventricular septal diameter was 8.6 mm ± 1.8, relative wall thickness was 0.37 ± 0.07 and indexed mass was 70 g/m^2^ ± 13.9. Left atrial volume indexed was 20.1 cm^2^/m^2^ ± 6.3, average septal e′ velocity was 11.4 cm/s ± 2.3, average lateral 15.9 cm/s ± 3.2, average E/e′ was 5.9 ± 1.5, average pulmonary artery systolic pressure was 22.5 mmHg ± 3.7. Average indexed right ventricular area for males was 11.2 cm^2^/m^2^ ± 2.1 and average fractional area change was 43.4% ± 7. One patient had grade 2 diastolic dysfunction, 7 (14%) had evidence of right ventricular dilatation, and 13 (26%) had evidence of left ventricular remodeling (Table 1).

**Table 1:** Demographic and clinical characteristics

Characteristic	All subjects (n = 50)
Age at HIV diagnosis (years)	28 (7.14)
Gender, male	48 (96)
Race/ethnicity	
African American	30 (60)
Caucasian	18 (36)
Other	2 (4)
Body-mass index (kg/m^2^)	25.4 (3.66)
Obese	4 (8)
Active smoker	10 (20)
Hypertension	4 (8)
Diabetes mellitus	–
Hyperlipidemia	5 (10)
Family history of cardiovascular disease	11 (22)
hs-CRP (mg/dL)	1.6 (2)
NT-proBNP (pg/mL)	19.6 (39.7)
Echocardiographic findings	
Left ventricle	
LV ejection fraction (%)	63.4 (6)
GLS (%)	− 0.31 (2.73)
LV remodeling^a^	13 (26)
LV diastolic dysfunction	1 (2)
Indexed LV mass (g/m^2^)	70 (13.9)
Interventricular septal diameter (mm)	8.6 (1.8)
Relative wall thickness	0.37 (0.07)
Right ventricle	
Indexed RV area (cm^2^/m^2^)	11.2 (2.1)
FAC (%)	43.4 (7)
RV dilatation^b^	7 (14)
LV diastolic function	
Indexed LA volume (cm^2^/m^2^)	20.1 (6.3)
Septal e′ velocity (cm/s)	11.4 (2.3)
Lateral e′ velocity (cm/s)	15.9 (3.2)
Average E/e′	5.9 (1.5)
Pulmonary artery systolic pressure (mmHg)	22.5 (3.7)
HIV disease characteristics	
CD4 count (cells/μL)	551 (225.1)
HIV viral load (copies/mL)^c^	112,585 (217,965)
AIDS diagnosis^d^	2 (4)
Time from last HIV negative test to first positive HIV test (days)	530 (265.2)
Estimated duration of infection prior to clinical evaluation (days)	304 (129.4)

All data expressed as number (%) or mean (± SD)

*hs*-*CRP* high-sensitivity C-reactive protein, *NT*-*proBNP* N-terminal probrain natriuretic peptide, *LV* left ventricular, *GLS* global longitudinal strain, *RV* right ventricular, *LA* left atrial, *FAC* fractional area change, *E/e′* early mitral inflow velocity/mitral annular early diastolic velocity

^a^Left ventricular remodeling was defined as relative wall thickness of > 0.42

^b^Determined by indexed diastolic RV area

^c^Viral load data excluded for 1 patient with viral load greater than the upper assay limit (> 1 × 10^7^ copies/mL)

^d^AIDS diagnostic criteria met by CD4 count < 200 cells/μL and or < 14%

#### Subgroup analysis

A subgroup analysis was performed for those patients found to have evidence of left ventricular remodeling. We did not find a statistically significant relationship between the presence of left ventricular remodeling and estimated duration of HIV infection (≥300 vs < 300 days; p = 0.339), CD4 count (≥ vs < 500 cells/μL; p = 0.197), HIV viral load (≥ vs < 100,000 copies/mL; p = 0.719), AIDS (p = 0.456) and traditional CVD risk factors (p > 0.05 for all) (Table 2).

**Table 2:** Subgroup analysis of patients with left ventricular remodeling

	All subjects (n = 50)	No LVR (n = 37)	LVR (n = 13)	*p* value^†^
Cardiovascular risk factors				
Body-mass index > 30 kg/m^2^	4 (8)	2 (5.4)	2 (15.4)	0.275
Active smokers	10 (20)	8 (21.6)	2 (15.3)	1
Hypertension	4 (8)	3 (8.1)	1 (7.7)	1
Hyperlipidemia	5 (10)	4 (10.8)	1 (7.7)	1
Family history of CVD	11 (22)	9 (24.3)	2 (15.4)	1
HIV characteristics				
HIV infection > 300 days	28 (56)	19 (51.4)	9 (69.2)	0.339
CD4 count < 500 cells/μL	20 (40)	17 (45.9)	3 (23.1)	0.197
HIV viral load > 100,000 copies/mL	13 (26)	9 (24.3)	4 (30.8)	0.719
AIDS	2 (4)	1 (2.7)	1 (7.7)	0.456

Patients with LVR compared to those without evidence of LVR. All data expressed as number (%)

*LVR* left ventricular remodeling

^†^Fisher’s exact test

### Discussion

To our knowledge this is the first study to describe subclinical echocardiographic abnormalities found in an actively seroconverting, ART naïve and asymptomatic young group of soldiers living with HIV. In the current study, asymptomatic, ART-naïve patients evaluated with echocardiography soon after HIV acquisition were not found to have evidence of systolic dysfunction or reduced global longitudinal strain. Some of the patients were found to have subclinical echocardiographic abnormalities such as RV dilatation and left ventricular remodeling however no statistically significant correlation was found with HIV-related risk factors identified. For this reason one may conclude that the uncontrolled viral replication, robust immune activation, and CD4 depletion that occurs shortly after HIV infection do not appear to directly cause asymptomatic clinically significant pathologic cardiac abnormalities. Prior to ART initiation, HIV-related cardiovascular disease has been attributed to direct myocyte invasion and myocardial toxicity [9]. However there is little known about the pathogenesis of the HIV-induced damage that takes place shortly after infection and before initiation of ART. Most of the studies in the ART era have focused on establishing the association between HIV infection and development of CVD in the aging population.

The current evidence supports that PLHIV on ART continue to have generalized immune activation and chronic inflammation which then leads to endothelial dysfunction and earlier development of subclinical cardiovascular disease when compared to the HIV-uninfected population [1, 10, 11]. Within the last decade there has been a focus on elucidating the prevalence of and understanding asymptomatic CVD among those with HIV infection using advanced noninvasive imaging technology [2, 3, 12, 13]. A meta-analysis of 9 European studies using echocardiography reports a prevalence of LV dysfunction (reduced EF) and diastolic dysfunction of 8.3% and 43.4% respectively among asymptomatic patients [14]. However, patients in these studies typically had a longer duration of HIV infection with the average time from HIV diagnosis to echocardiographic evaluation of 8 years and 98% of the patients were on ART for an average of 5 years. This is a major difference from our study as none of the patients in our study were being treated with anti-retroviral therapy or any other medication at the time of echocardiographic evaluation. An observational single-institution study of 41 ART-naïve asymptomatic newly diagnosed HIV patients in Greece found no echocardiographic evidence of reduced LVEF, diastolic dysfunction or evidence of elevated pulmonary pressures. However, evidence of GLS abnormalities was observed in all the patients studied and the authors concluded that HIV infection itself has myocardial toxicity and causes subclinical systolic dysfunction. Unfortunately, this study was did not account for the duration of HIV infection and heterogeneity of the population studied [15]. In contrast, all the patients in our study were previously healthy and were documented HIV seroconverters with an average of 10 months duration of infection prior to echocardiography. It is possible that our patients underwent echocardiography before the development of expected HIV-related GLS abnormalities described in other studies. Based on our results we suggest that the LVR findings in our population are related to reversible physiologic adaptation to physical activity rather than a pathologic effect related to the HIV infection itself. However, it must be noted that the prevalence of left ventricular remodeling and ventricular dilatation seen in our study population appears to be slightly higher than what has been previously reported in healthy athletes [16–19].

In conclusion, the main echocardiographic abnormality observed in our study group was left ventricular remodeling. Our study did not find a clear relationship between LVR and duration, degree of viremia or immunosuppression in these asymptomatic early after seroconversion. We believe our findings provide new insight about the relationship between uncontrolled viral replication and cardiovascular health in asymptomatic PLHIV.

## Limitations

The main limitation of our study is the lack of an age-matched control group in order to account for the echocardiographic abnormalities observed. Even though the clinical significance of asymptomatic LVR depends on the physical activity level of each individual, our study did not quantify the physical activity level of each patient. We believe further longitudinal studies are warranted to better determine the optimal CVD screening and risk assessment strategies for asymptomatic PLHIV.

## Data Availability

The datasets generated and/or analyzed during the current study are not publicly available due to military institutional policies but are available from the corresponding author on reasonable request.

## Abbreviations

ART: anti-retroviral therapy
CD4: serum CD4 cell count
CVD: cardiovascular disease
EF: systolic ejection fraction
GLS: global longitudinal strain
HIV: human immunodeficiency virus
LVR: left ventricular remodeling
hs-CRP: high-sensitivity C reactive protein
NT-proBNP: N-terminal probrain natriuretic peptide
PLHIV: people living with HIV
RV: right ventricle
SAMMC: San Antonio Military Medical Center
TTE: transthoracic echocardiogram
USAF: United States Air Force
VL: serum HIV viral load
